# Non-linear Membrane Properties in Entorhinal Cortical Stellate Cells Reduce Modulation of Input-Output Responses by Voltage Fluctuations

**DOI:** 10.1371/journal.pcbi.1004188

**Published:** 2015-04-24

**Authors:** Fernando R. Fernandez, Paola Malerba, John A. White

**Affiliations:** Department of Bioengineering, University of Utah, Salt Lake City, Utah, United States of America; Imperial College London, UNITED KINGDOM

## Abstract

The presence of voltage fluctuations arising from synaptic activity is a critical component in models of gain control, neuronal output gating, and spike rate coding. The degree to which individual neuronal input-output functions are modulated by voltage fluctuations, however, is not well established across different cortical areas. Additionally, the extent and mechanisms of input-output modulation through fluctuations have been explored largely in simplified models of spike generation, and with limited consideration for the role of non-linear and voltage-dependent membrane properties. To address these issues, we studied fluctuation-based modulation of input-output responses in medial entorhinal cortical (MEC) stellate cells of rats, which express strong sub-threshold non-linear membrane properties. Using *in vitro* recordings, dynamic clamp and modeling, we show that the modulation of input-output responses by random voltage fluctuations in stellate cells is significantly limited. In stellate cells, a voltage-dependent increase in membrane resistance at sub-threshold voltages mediated by Na^+^ conductance activation limits the ability of fluctuations to elicit spikes. Similarly, in exponential leaky integrate-and-fire models using a shallow voltage-dependence for the exponential term that matches stellate cell membrane properties, a low degree of fluctuation-based modulation of input-output responses can be attained. These results demonstrate that fluctuation-based modulation of input-output responses is not a universal feature of neurons and can be significantly limited by subthreshold voltage-gated conductances.

## Author Summary

The membrane voltage of neurons *in vivo* is dominated by noisy “background” fluctuations generated by network-based synaptic activity from nearby cells. It has been speculated that membrane voltage fluctuations in neurons play an important role in scaling the relationship between input amplitude and spike rate response. For this to be true, neuronal spike input-output behavior must be sensitive to physiological membrane voltage fluctuations. Using a combination of single cell recordings and modeling, we investigated the mechanisms through which voltage fluctuations modulate neuronal input-output responses. We find that neurons that express an increase in membrane input resistance with depolarization show low levels of noise-mediated modulation of input-output responses due, in part, to voltage trajectories that suppress the likelihood of generating a spike in response to random current input fluctuations. Hence, non-linear membrane properties arising from certain types of voltage-gated conductances limit noise-based modulation of neuronal input-output responses.

## Introduction

Membrane voltage in cortical neurons is dominated by fluctuations mediated by random synaptic activity [1–4]. Because probabilistic threshold crossings associated with fluctuations lower spike threshold, enabling spike response to otherwise sub-threshold inputs [5,6], it has been hypothesized that background activity amplifies neuronal sensitivity, and in doing so permits fluctuations to modify the input-output functions of neurons [7–12]. Consistent with this hypothesis, recordings *in vivo* often show a large variance in interspike intervals [13,14]. Spectral properties of voltage fluctuations are also correlated with different cognitive states, lending support to the idea that fluctuations play an important role in modulating spike output [4,15–17]. Finally, computational models suggest that neurons are sensitive to transient inputs and modulate their input-output function in response to changes in the size of membrane voltage fluctuations [10,18–20].

For two reasons, however, it is not clear that results of strong effects of membrane-potential fluctuations on input-output relationships hold in general. First, data supporting a strong relationship come from only a few types of neurons [8,11,21–23]. Second, even these restricted studies have shown significant variability in the magnitude of the effect [21,23–25]. These observations indicate a possible complex relationship between membrane voltage fluctuations and neuronal input-output modulation. Modulation of input-output responses is likely influenced by numerous factors, including sub-threshold voltage-dependent properties present in neurons. For example, the negative slope conductance associated with Na^+^ current, which increases membrane resistance in close proximity to spike threshold [26], has been shown to reduce neuronal responsiveness to high frequency voltage fluctuations in model neurons [27].

To examine how non-linear membrane properties determine the degree of fluctuation-based modulation of input-output responses in neurons, we recorded from MEC stellate cells. These neurons express strong non-linear membrane properties at sub-threshold voltages and are characterized by a voltage-dependent change in membrane resistance [28–30]. Like other cortical neurons, *in vivo* recordings of stellate cells have established the presence of large membrane voltage fluctuations that have the potential to influence input-output responses [31,32].

Using standard measures of spike output in the form of spike frequency-current and spike-probability curves, as well as analysis of spike generation in an exponential leaky integrate-and-fire model, we investigated the biophysical factors regulating the ability of voltage fluctuations to modify stellate cell input-output measures. We find that non-linear membrane properties associated with increased membrane resistivity at sub- and peri-threshold voltages reduce fluctuation-based modulation of input-output responses. Overall, our results indicate that fluctuation-based modulation of neuronal input-output responses can be very low, with limited scaling of spike output via changes in noise and conductance levels.

## Results

### Stellate cell input-output functions are modulated weakly by membrane voltage fluctuations

To investigate the modulation of input-output responses by membrane voltage fluctuations in MEC stellate cells, we started by quantifying fluctuation-induced changes in commonly used measures of neuronal input-output responses. These include the slope (gain) and rheobase of frequency-current (*f-I*) and spike-probability curves. Voltage fluctuations were generated using current-based fluctuations that were constructed using low-pass filtered white noise (see Methods). For each cell, we recorded a short trial period in which the current input fluctuation amplitude was adjusted to maintain a standard deviation (SD) in output voltage at rest (-75 mV, corrected for the electrode’s junction potential) of approximately 2.5 mV (2.41 ± 0.1 mV), a value commonly observed *in vivo* [33]. Controlling for the SD of voltage fluctuations was essential since the intrinsic properties of neurons are voltage-dependent and a fair comparison across different cells, conditions and models require that the SD of membrane voltage be constant. Furthermore, previous work addressing similar issues has controlled fluctuation sizes in terms of the SD of membrane voltage and used similar values [8,9,19,23,24,34,35],

For *f-I* curves, spike frequency was determined using only the first three inter-spike intervals in order to avoid complications arising from the interaction between the time scale of voltage fluctuations and spike frequency adaptation [21,23]. Gain was calculated individually for each cell using the slope of a linear fit (r^2^: 0.64 to 0.96, mean: 0.89 ± 0.02) to the *f-I* relationship, while rheobase was measured as the current required to elicit a minimum of 3 inter-spike intervals from a holding voltage of -75 mV.

**Fig 1:**
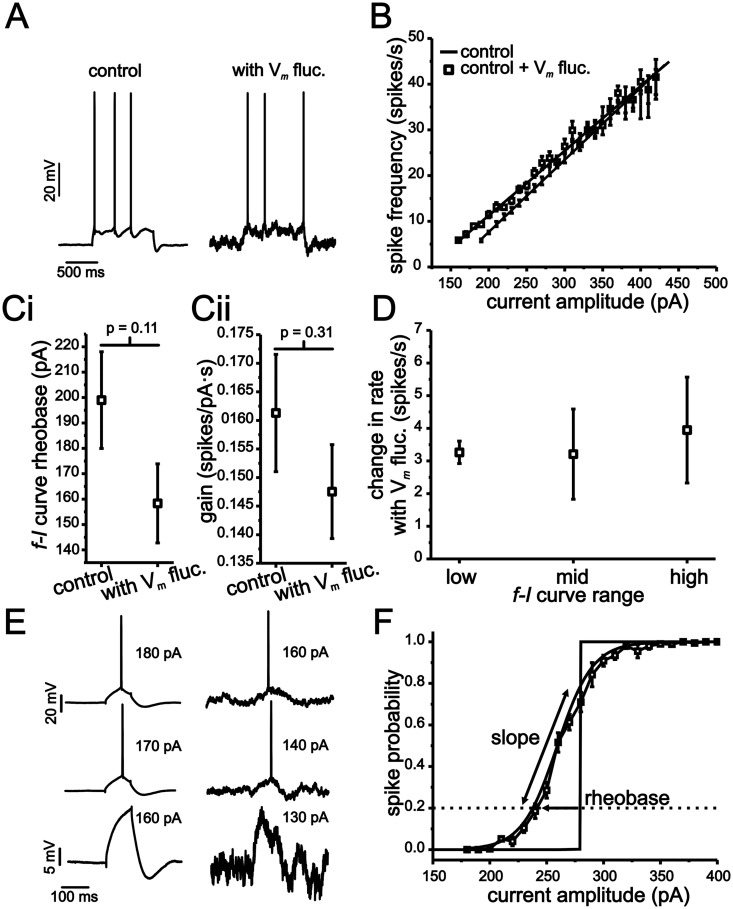
MEC stellate cell input-output relationships express a low sensitivity to membrane voltage fluctuations. (A) Representative examples of stellate cell voltage response to 1 s long current steps with (right panel) and without (left panel) membrane voltage fluctuations. (B) Plot of average *f-I* relationships under conditions outlined in A. (C) Mean leftward shifts in rheobase (i) and gain (ii) in *f-I* curves resulting from the introduction of membrane voltage fluctuations. (D) Change in firing rate resulting from membrane voltage fluctuations for low, mid and high spike rate regions of the *f-I* relationship. For each region, changes in firing rate were calculated using the difference in an individual cell’s firing rate resulting from the introduction of current input fluctuations. (E) Representative examples of stellate cell voltage response to 100 ms long current steps of different amplitudes with and without membrane voltage fluctuations. (F) Average spike-probability curve in the presence of membrane voltage fluctuations. The vertical line indicates average rheobase in the absence of voltage fluctuations for each condition.

As shown in Fig 1B, stellate cell *f-I* curves were only modestly influenced by the introduction of membrane voltage fluctuations. The addition of fluctuations generated a small, but non-significant, leftward shift in rheobase (Fig 1Ci; 199 ± 20 pA vs. 158 ± 20 pA, p = 0.11, n = 20, 18). As with rheobase, the addition of voltage fluctuations did not generate a significant reduction in the *f-I* curve gain (Fig 1Cii; 0.161 ± 0.10 spikes/ pA s. vs. 0.148 ± 0.08 spikes/pA s, P = 0.31, n = 20, 18). To quantify potential changes in the *f-I* curve more carefully, we also measured the effects of voltage fluctuations on firing rate within discrete regions of the *f-I* curve (Fig 1D; low, mid and high). Previous modeling and experimental work has shown that random voltage fluctuations induce the largest increase in firing rate in the low spike rate region of the *f-I* curve, near the transition between rest and firing [8–10,19,20,24]. For our data, the low region was defined individually for each cell as the frequency of spike discharge at rheobase, while the mid and high regions corresponded to current values eliciting 15 spikes/s and 25 spikes/s more than the initial frequency, respectively. For each cell, we measured the change in firing rate brought about by voltage fluctuations for low, mid and high current input regions relative to the same cell’s *f-I* curve acquired without fluctuations. Differences in spike rate were small but changed significantly in the low and mid regions of the *f-I* curve. For the low region of the *f-I* curve, fluctuations induced an increase of 3.3 ± 0.34 spikes/s (Fig 1D; P <0.001, n = 18), while in the mid region, these values were 3.2 ± 1.4 spikes/s (P = 0.03, n = 18).

Next, we measured the effects of voltage fluctuations on spike-probability curves. Unlike the *f-I* curve, which requires repetitive spike generation at each current step size, a spike-probability curve quantifies the probability of generating just a single spike within a given time window (100 ms in our case, Fig 1E and 1F). In the absence of membrane voltage fluctuations, the transition from a probability of zero to one in spike discharge occurred almost always within a single current step (Fig 1F; vertical line). For experiments without artificial voltage fluctuations, the current amplitude associated with this transition point was defined as rheobase. The addition of voltage fluctuations smoothed the relationship between spike probabilities and current steps such that data points could be fit with a sigmoid function (Fig 1F; Boltzmann fit, r^2^ >0.96). In the presence of voltage fluctuations, rheobase was defined as the current needed to establish a 0.2 probability (P_0.2_) in generating a single spike, while the slope was quantified using the “*k*” term in the Boltzmann function (see Methods). As shown, voltage fluctuations induced a leftward shift in rheobase relative to conditions without fluctuations (Fig 1F; 56 ±5.6 pA, P <0.001, Student t-test, n = 20) and resulted in spike-probability curves with an average slope of 26 ± 8.4 pA (mean ± s.e.m).

Overall, modifications of stellate cell *f-I* curves by voltage fluctuations were small compared to previous work in other neurons [8,9,23,24,35], with no significant modulation of *f-I* curve gain across the population and relatively small changes in initial firing rate. In comparison, past work using similarly sized voltage fluctuations has reported reductions in gain of up to 50% [8,9]. Nevertheless, stellate cells do show some degree of fluctuation-mediated modulation of input-output responses as indicated by a shiftand smoothing of the spike-probability curve.

### Stellate cells express significant non-linear membrane properties leading up to spike threshold

Given both theoretical and experimental work supporting a strong modulatory role for membrane voltage fluctuations, we were interested in what factors control and limit fluctuation-based changes of input-output responses in stellate cells. As a potential cause for the limited fluctuation-based modulation of input-output responses, we considered the role of non-linear membrane properties leading up to spike threshold. In simple models, realistic spike generation dynamics have been shown to reduce the likelihood of spike response to rapid voltage fluctuations [27,36]. We hypothesized that an extension of this effect over a much larger sub-threshold voltage region than has been previously considered could significantly reduce fluctuation-based modulation of input-output responses.

**Fig 2:**
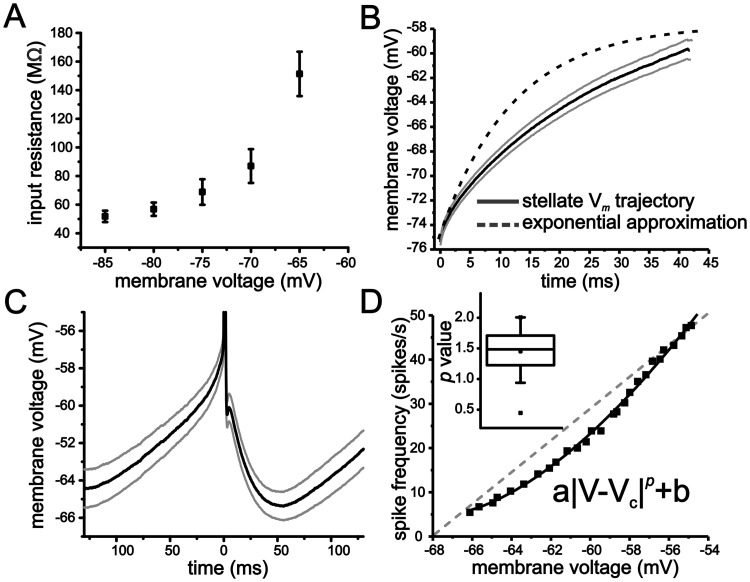
Stellate cells express non-linear membrane properties that shape the voltage trajectory leading up to spike threshold. (A) Average steady-state input resistance as a function of membrane voltage. Measures were taken in voltage-clamp using a 100 ms duration 5 mV step. (B) Average membrane voltage trajectory (grey lines indicate SEM) associated with approach from rest to first spike. For comparison, the exponential approximation using the membrane time constant measures taken at -75 mV is also shown. (C) Average membrane voltage trajectory (grey lines indicate SEM) associated with the interspike interval. (D) Representative example of an *f-V* curve from a stellate cell (solid line). Line indicates fit to a power-law function of the form shown in panel inset. Inset also shows box-plot of mean exponent (*p*) value for the power-law function fit.

To first establish the presence of sub-threshold non-linear membrane properties in stellate cells, we quantified membrane input resistance between -85 mV and -65 mV. At each holding voltage, membrane resistance was measured in voltage-clamp using a 5 mV voltage step of 100 ms duration. Depolarizing stellate cells led to a progressive increase in steady-state membrane input resistance (Fig 2A; one-way ANOVA, P <0.001, n = 12). Membrane resistance nearly tripled over a 20 mV range, increasing from 51.8 ± 3.9 MΩ at -85 mV to 151.4 ± 15.5 MΩ at -65 mV (Fig 2A). We should note that resistance also kept increasing with additional depolarization to the extent that very small voltage steps (~ 1 mV) often elicited spikes at levels more depolarized than -65 mV and prevented an accurate measure of membrane resistance at these voltage values.

Next, we analyzed average membrane voltage trajectories leading up to spike threshold. For current inputs eliciting small changes in voltage (5 mV), the resulting voltage trajectory was fit accurately with an exponential function and used to extract the membrane time constant near -75 mV (12.0 ± 0.9 ms, n = 19). In contrast, the membrane voltage trajectory to the first spike (for a 50 ms first spike latency) from a holding voltage of -75 mV (~ 15 mV range) could not be fit with an exponential function due to the more linear profile of the trajectory (Fig 2B). Similarly, the average voltage trajectory between the trough of the afterhyperpolarization (AHP) and spike threshold during continuous firing (~ 4 Hz) was not exponential (Fig 2C). Thus, the voltage trajectories to spike threshold starting either from resting voltages or the trough of the AHP were relatively linear compared to that expected from our measures of the membrane time constant at—75 mV.

To assess how changes in spike rate relate to changes in the average voltage associated with spike trajectories at different spike frequencies, we quantified the relationship between spike frequency and mean voltage (*f-V*). As with the *f-I* curves, spike rate for the *f-V* curve was taken from the first three inter-spike intervals, with membrane voltage values calculated as the mean during the same period of time. Surprisingly, we found that *f-V* curves were non-linear and could be fit with a power-law function (Fig 2D; mean r^2^ = 0.95 ± 0.02, range: 0.7–0.99) using an exponent (*p*) of 1.45 ± 0.08 (Fig 2D; n = 19, range: 0.45–2.0, 17/19 had *p* values >1). Cell *f-V* curves were also shallow, with an average slope across the firing range of 4.5 ± 0.4 spikes/mV s (n = 19). Contrary to previous assumptions [12,37], therefore, neuronal *f-V* curves can express significant power-law scaling in the absence of any fluctuation-based smoothing. In summary, our measures of membrane resistance, voltage trajectories, and *f-V* curves indicate that stellate cells express significant sub- and peri-threshold nonlinearities.

### A gradual increase in membrane resistance is critical to reduced fluctuation-based modulation of input-output responses in an eLIF model

To understand the biophysical mechanisms and consequences of a voltage-dependent membrane resistance over a large region of sub-threshold voltage, we started by studying the effects of fluctuation-based modulation of input-output behavior in a simplified model of spike generation in the form of an exponential leaky integrate-and-fire (eLIF) model [27]. This model has the advantage of incorporating important non-linear membrane properties associated with spike threshold using a small set of parameters that are related to physiological measures (e.g. voltage-dependent sub-threshold membrane resistance).

**Fig 3:**
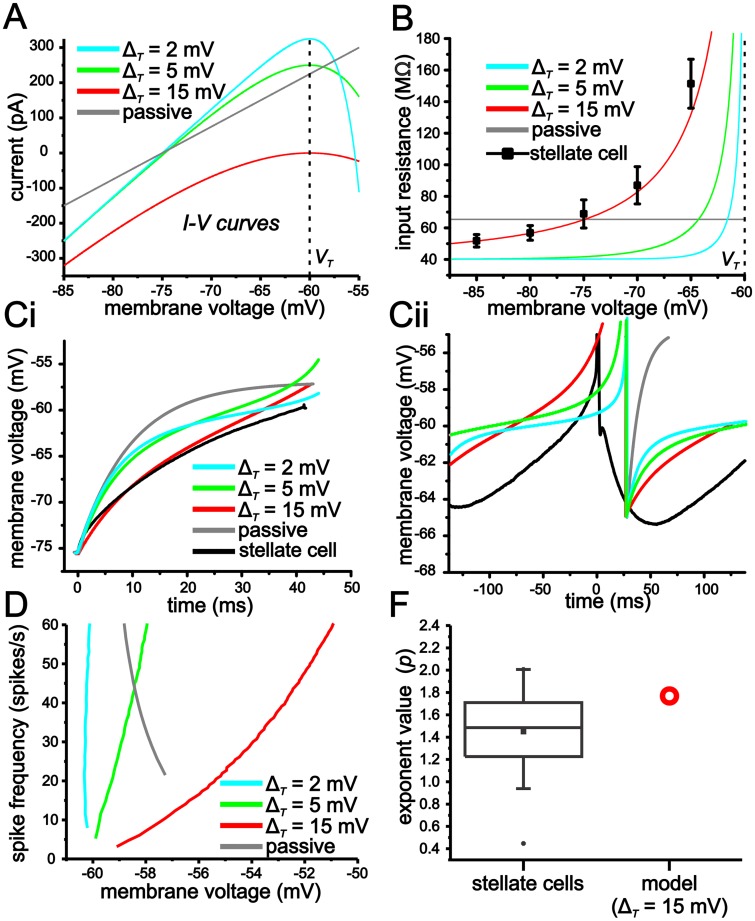
An eLIF model with a large Δ_*T*_ value accurately captures membrane voltage properties observed in stellate cells. (A) Plot of *I-V* curves from eLIF models using different Δ_*T*_ (2, 5, 15 mV) values, as well as a completely passive model. (B) Plot of input resistance as a function of membrane voltage for models outlined in *A*. Note, average stellate cell values are best matched by an eLIF using a Δ_*T*_ value of 15 mV. (C) Voltage trajectories for initial spike approach (i) and interspike interval (ii) for models outlined in A. For comparison, average stellate cell trajectories are also shown. (D) *f-V* curves for models outlined in A. (F) Plot comparing experimental values for power-law function exponent in stellate cells and eLIF model using a Δ_*T*_ of 15 mV.

The spike slope factor (Δ_*T*_) in the eLIF determines the change in slope of the membrane voltage-current (*I-V*) curve as membrane voltage approaches spike threshold (V_*T*_—Fig 3A). The exponential term models the increase in membrane resistance associated with an increase in Na^+^ conductance activation and spike generation. With small Δ_*T*_ values, the *I-V* curve slope (membrane resistance) changes abruptly as the system approaches V_*T*_ (Fig 3A). Conversely, with large Δ_*T*_ values, the change in slope is more gradual. Thus, for a small Δ_*T*_ value (e.g. 2 mV), the *I-V* curve is largely linear with the exception of a small voltage range in the immediate vicinity of V_*T*_. As Δ_*T*_ becomes larger, however, membrane resistance increases gradually over a relatively large span of sub-threshold membrane voltage values. We found that a Δ_*T*_ value of 15 mV best matched experimental values of steady-state membrane input resistance observed in stellate cells (Fig 3B). We should note that a value 15 mV for Δ_*T*_ is large compared to that implemented in previous work (0.8 m V to 6 mV) using an eLIF model to study cortical neurons [27,38,39].

We started by comparing stellate cell membrane voltage trajectories to those generated in the eLIF model using Δ_*T*_ values of 2, 5 and 15 mV. For comparison, we also included a completely passive model (i.e. standard leaky integrate-and fire model-LIF) consisting of a linear conductance term (15 nS, -75 mV reversal) and an artificial threshold (-55 mV). For all models, membrane voltage was reset (V_*r*_) to -65 mV after crossing threshold.

The eLIF model with a Δ_*T*_ of 15 mV does a substantially better job in reproducing the more linear approach associated with the initial approach to spiking from -75 mV (Fig 3Ci). Likewise, for the interspike interval voltage trajectory between the AHP trough and spike threshold during repetitive spike discharge (~4 Hz), a Δ_*T*_ value of 15 mV best captures the linear trajectory leading up to spike threshold observed in stellate cells (Fig 3Cii). As Δ_*T*_ is reduced, the voltage trajectories approaching spike threshold become more exponential and qualitatively more like the passive model (Fig 3C).

Given the differences in voltage trajectories both in the initial approach to spiking and the interspike voltage trajectories, we were interested in how mean membrane voltage scaled with spike rate (*f-V*) in each of the models. For the passive model, the *f-V* curve is steep and has a negative slope. In the active models, both the slope and shape changes considerably with different Δ_*T*_ values. As the value of Δ_*T*_ is increased from 2 mV to 15 mV, the *f-V* curve slope goes from negative and steep to shallow and positive (Fig 3D). Furthermore, over the range of 1–60 spikes/s, the *f-V* curve of the eLIF using a value of Δ_*T*_ of 15 mV can be accurately fit with a power-law function with an exponent of 1.78, which is within the range of values observed experimentally for stellate cells (Fig 3F).

To quantify the modulation of input-output responses by voltage fluctuations in the models, we compared results in the eLIF model using Δ_*T*_ values of 2 mV and 15 mV. We delivered the same current input fluctuations as in stellate cells, and maintained voltage fluctuations with a SD of 2.5 mV at -75 mV. Previous studies have also established that increasing membrane conductance via a variety of mechanisms, which include shunting inhibition, balanced synaptic conductances or simply increasing membrane leak, facilitates modulation of the *f-I* curve using similarly sized voltage fluctuations [8–10,22]. In addition, as a result of the different slopes of the exponential terms, the eLIF models using Δ_*T*_ values of 2 mV and 15 mV have different input resistance values at -75 mV (Fig 3B), a property that may account for potential differences in fluctuation-based modulation of input-output responses. For these reasons, a separate shunt or leak conductance (*g*_*L*_) of 15 nS was introduced in each of the models to test the effect of increasing membrane conductance on input-output modulation.

**Fig 4:**
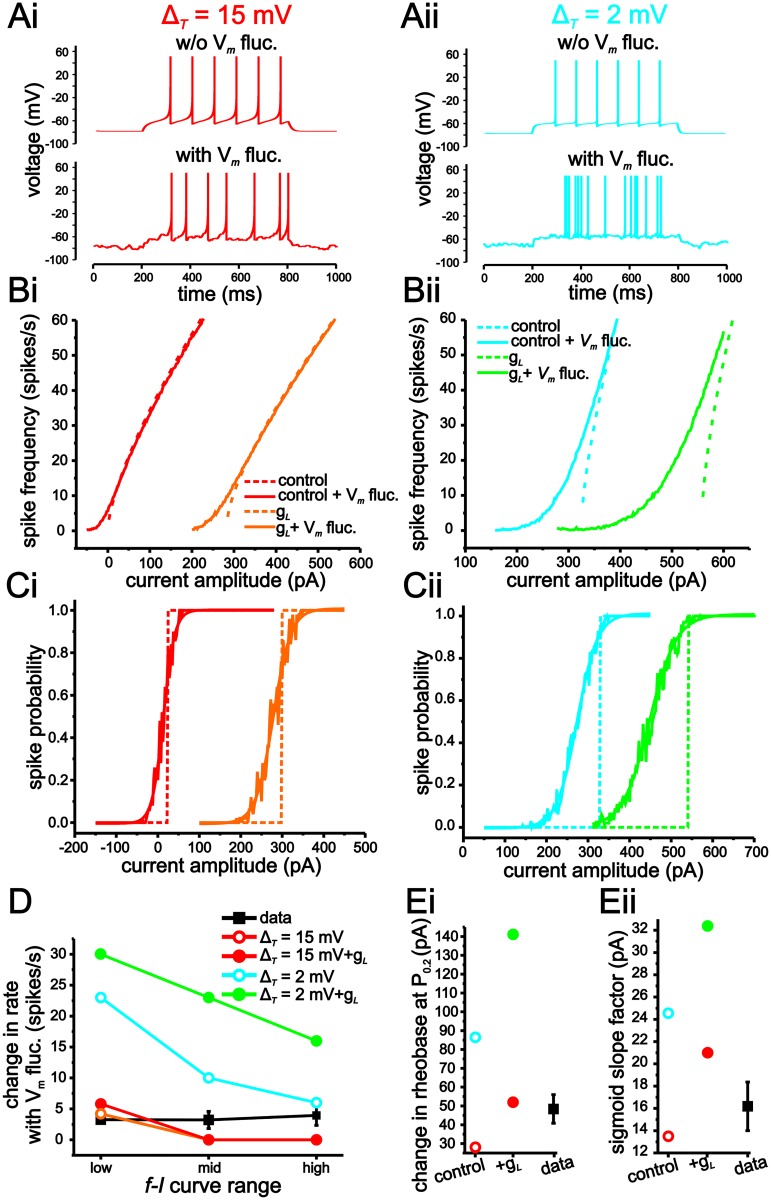
An eLIF model using a Δ_*T*_ value of 15 mV generates limited modulation of input-output responses by membrane voltage fluctuations. (A) Example traces of eLIF models implemented with Δ_*T*_ = 15 mV (i) and 2 mV (ii). (B) *f-I* curves in eLIF models with Δ_*T*_ = 15 mV (i) and 2 mV (ii) with or without membrane voltage fluctuations under baseline (black) or increased membrane conductance (*g*_*shunt*_-grey). (C) Spike-probability curves in eLIF models with Δ_*T*_ = 15 mV (i) and 2 mV (ii) with or without membrane voltage fluctuations under baseline (black) or increased membrane conductance (*g*_*shunt*_-grey). (D) Changes in firing rate induced by membrane voltage fluctuations for low, mid and high regions of the *f-I* curves for each model. For comparison, average stellate cell values are also shown. (E) Plots of changes in rheobase (i) and sigmoid slope factor (ii) in spike-probability measures associated with the introduction of membrane voltage fluctuations for each model.

Fluctuation-based modulation of both the *f-I* and spike-probability curves are substantially larger using a value for Δ_*T*_ of 2 mV than with 15 mV (Fig 4). Consistent with previous computational and experimental results [7–10], increasing membrane conductance using shunting inhibition increases fluctuation-induced modulation of input-output responses in both models, albeit the effect is much larger when Δ_*T*_ = 2 mV (Fig 4). With Δ_*T*_ = 2 mV, fluctuation-induced increases in initial spike firing rates are 23 spikes/s and 30 spikes/s under baseline and with increased membrane conductance, respectively (Fig 4Bii–4D). In comparison, with Δ_*T*_ = 15 mV, these value are only 4.2 spikes/s and 5.8 spikes/s (Fig 4Bi–4D). Similarly, for spike-probability curves, voltage fluctuations result in larger leftward shifts and smoothing under both conductance conditions with Δ_*T*_ = 2 mV (Fig 4C–4E). As a result, the changes in firing rate, rheobase and gain induced by membrane voltage fluctuations correspond more closely to those observed in stellate cells when Δ_*T*_ is set to 15 mV (Fig 4D and 4E).

**Fig 5:**
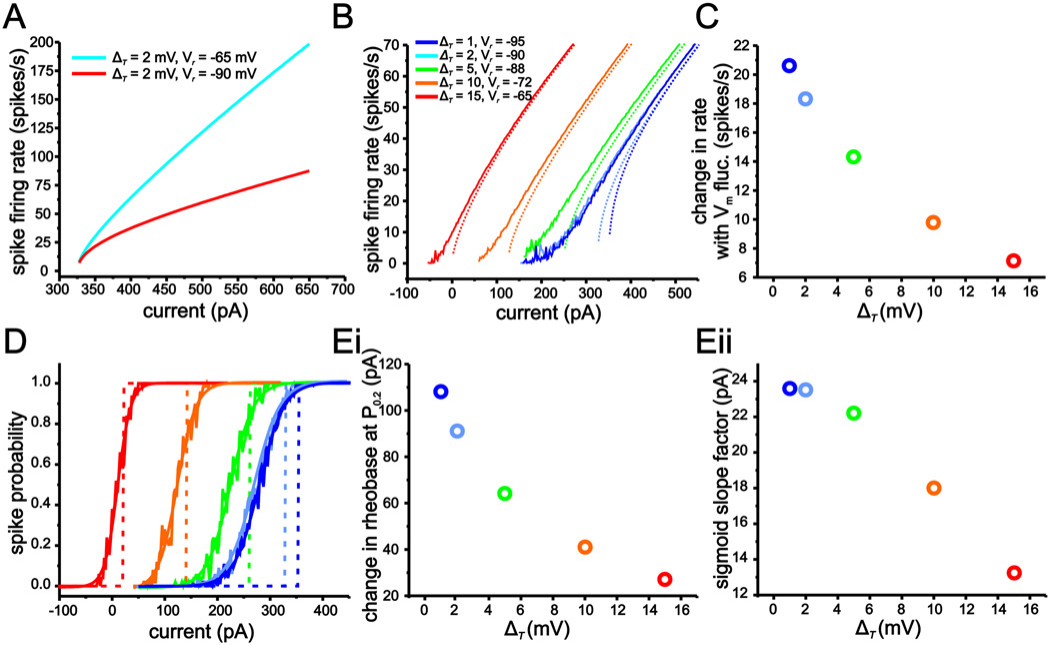
Reducing Δ_*T*_ in the eLIF model gradually increases modulation of input-output responses by membrane voltage fluctuations. (A) eLIF model *f-I* curve gain is reduced with more a negative V_*r*_ value. (B) Comparison of *f-I* curves for different Δ_*T*_ values with (solid lines) and without (dash lines) membrane voltage fluctuations. Note, more negative V_*r*_ values were used to maintain the same gain with smaller Δ_*T*_ values. (C) Changes in the initial firing rate of *f-I* curves induced by membrane voltage fluctuations for the eLIF model using Δ_*T*_ values. (D) Comparison of spike-probability curves for different Δ_*T*_ values with (solid lines) and without (dash lines) membrane voltage fluctuations. (E) Plots of changes in rheobase (i) and sigmoid slope factor (ii) on spike-probability measures associated with the introduction of membrane voltage fluctuations for eLIF models using different Δ_*T*_ values.

Decreasing the Δ_*T*_ also leads to an increase in the gain of the *f-I* curve (Fig 4B). Consequently, the increase in fluctuation-induced modulation of the *f-I* curves with lower Δ_*T*_ values could result from an intrinsic higher gain value that provides greater sensitivity to changes in current input fluctuations. Prior work has established that decreasing the V_r_ value has a divisive effect on gain [40]. To eliminate the influence of higher gain values, therefore, we incrementally decreased the V_*r*_ value to decrease gain when Δ_*T*_ was small (Fig 5A). Using this approach, we could maintain gain approximately equal across different Δ_*T*_ values and test if differences in fluctuation-induced smoothing of the *f-I* curve are entirely due to changes in intrinsic gain (Fig 5A and 5B). Although compensating for gain through changes in V_*r*_ decreases fluctuation-based modulation of the *f-I* curve, lower Δ_*T*_ values still result in greater smoothing and increases in the initial firing rate of the *f-I* curve (Fig 5C).

For the sake of completeness we also quantified changes in rheobase and slope of spike probabilities curves induced by the introduction of voltage fluctuations (Fig 5D). For these measures the choice of V_*r*_ has no impact. As shown, a gradual decrease in Δ_*T*_ from 15 mV to 1 mV results in a gradual increase in both the ability for fluctuations to shift rheobase and smooth the spike-probability curves (Fig 5E).

### Limited fluctuation-based modulation of input-output responses can be reproduced in a conductance-based model using Hodgkin and Huxley formulism

**Fig 6:**
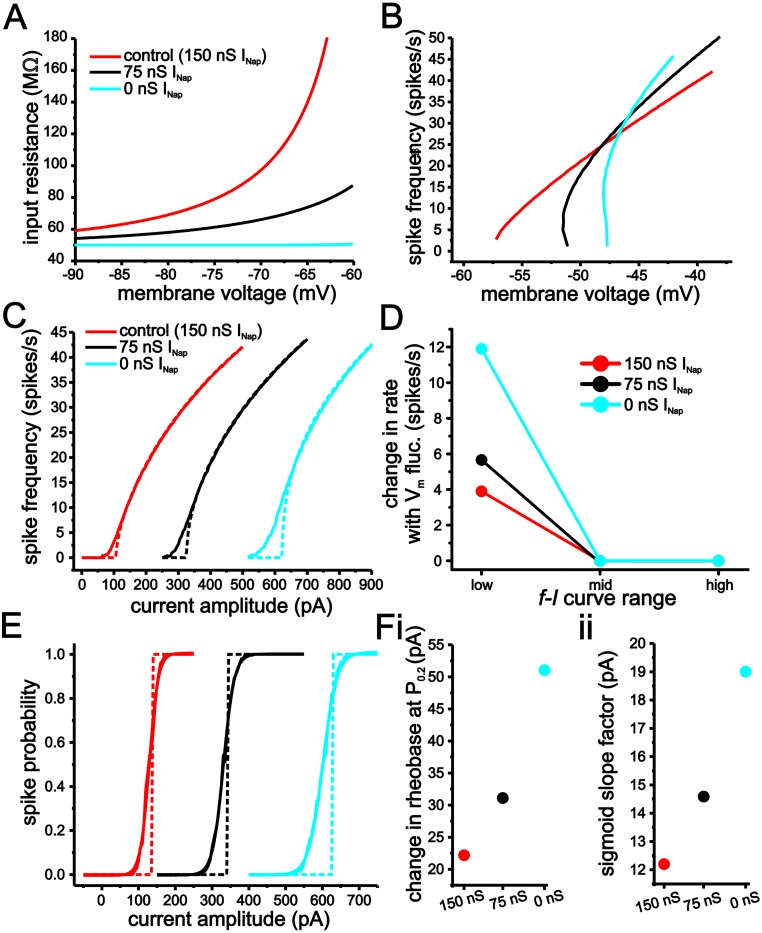
Reduction of persistent Na^+^ current in an H-H formulism-based conductance model increases modulation of input-output responses by voltage fluctuations. (A, B) Plot of steady-state membrane input resistance (A) and spike frequency (B) as a function of membrane voltage in the model for 3 different levels of I_Nap_ (150 nS, 75 nS and 0 nS). (C) *f-I* relationships in the H-H based model using 150 nS (red), 75 nS (black) and 0 nS (cyan) with (solid line) or without (dashed line) membrane voltage fluctuations. (D) Change in firing rate resulting from membrane voltage fluctuations for low, mid and high spike rate regions of the *f-I* relationship. (E) Spike probability curves in the H-H based model using 150 nS (red), 75 nS (black) and 0 nS (cyan) with (solid line) or without (dashed line) membrane voltage fluctuations. (F) Plots of changes in rheobase (i) and sigmoid slope factor (ii) on spike-probability measures resulting from the introduction of membrane voltage fluctuations for the H-H based model using different levels of I_Nap_.

Although the eLIF model using a large Δ_*T*_ generates a very good match to the experimental results attained in stellate cells, we were interested if a more biologically plausible model using standard Hodgkin and Huxley (H-H) formulism could also reproduce our experimental results. For our H-H-based model, we started with a non-inactivating Na^+^ conductance (I_Nap_) that generates the gradual increase in membrane input resistance at sub-threshold membrane voltages observed in stellate cells (Fig 6A). For spiking currents, we used a standard transient Na^+^ current coupled with a slower K^+^ current (see Methods). Note that the rheobase values in the conductance-based model differed from the eLIF model because the voltage threshold for spiking was more depolarized than for the eLIF.

As expected, reducing the magnitude of I_Nap_ decreases the gradual increase in subthreshold membrane input resistance (Fig 6A). Further, the reduction of I_Nap_ increases the slope of the *f-V* curve (Fig 6B). Consequently, the presence of I_Nap_ drives the model neuron to generate an *f-V* more similar to the eLIF model using a Δ_*T*_ = 15 mV, while without I_Nap_, the *f-V* curve is more similar to when Δ_*T*_ = 2 mV.

To evaluate the role of sub-threshold resistance on fluctuation-based modulation, we varied the conductance magnitude of I_Nap_. As before, we added current input fluctuations so as to generate voltage fluctuations with a 2.5 mV STD at -75 mV. For both *f-I* (Fig 6C and 6D) and spike probability (Fig 6E and 6F) measures, the reduction of I_Nap_ leads to a progressive increase in the ability for membrane voltage fluctuations to modulate the input-output responses. Thus, a standard H-H conductance-based model can reproduce experimental results and observations from the eLIF model.

### Large Δ_T_ values reduce modulation of input-output responses through voltage fluctuations by slowing membrane voltage

**Fig 7:**
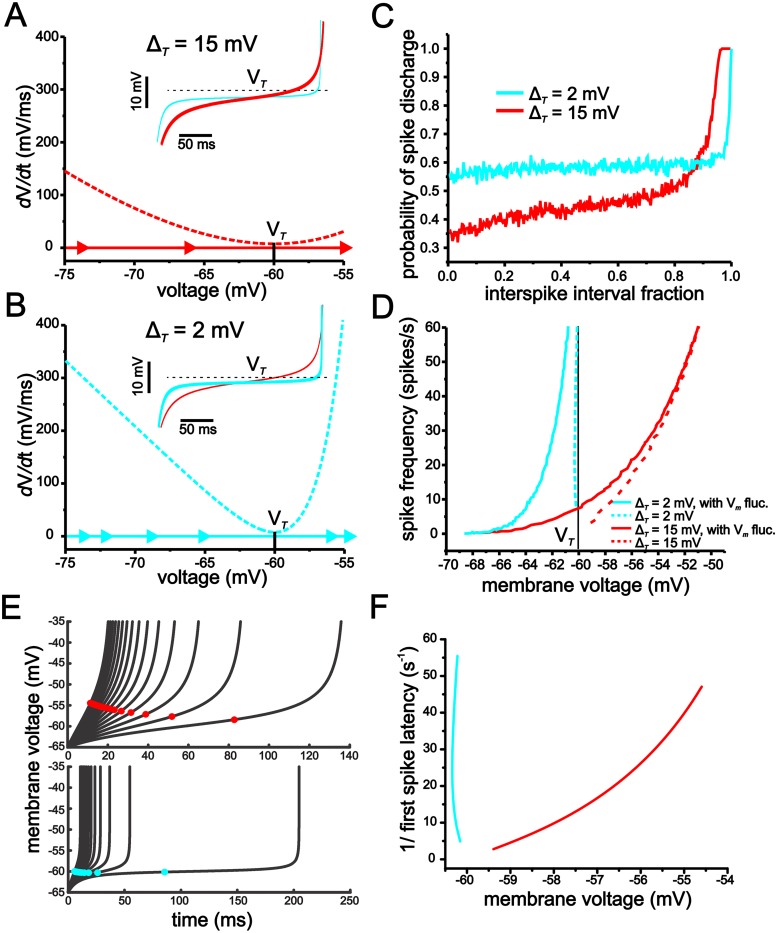
The Δ_*T*_ value determines the sensitivity to membrane voltage fluctuations in the eLIF model by setting the rate of change in membrane voltage and spike discharge probability during the approach to spike threshold. (A, B) Phase-plane plots for the eLIF model using Δ_*T*_ values of 15 mV (A) and 2 mV (B). Dotted lines indicate the membrane voltage derivative (*d*V*/d*t) value, with sold lines and arrows indicating trajectory and relative rate of change (distance between arrows). A smaller Δ_*T*_ value results in a faster rate of change in membrane voltage during the approach to voltage threshold (minima of *d*V*/d*t line, V_*T*_). Insets show membrane voltage trajectory associated with phase-plane plots in *A* and *B*. (C) Plot of spike-probability in response to membrane voltage fluctuations during a ~ 4Hz interspike interval for the eLIF model using a Δ_*T*_ value of 15 mV (red) and 2 mV (blue). (D) Plot of the *f-V* curves for the eLIF model using a Δ_*T*_ value of 15 mV (red) and 2 mV (blue) with (solid lines) and without (dashed lines) membrane voltage fluctuations. (E) Plot of voltage trajectory for different levels of applied current for the eLIF model using Δ_*T*_ values of 15 mV (top) and 2 mV (bottom). Model was solved using an initial condition of -65 mV and stopped once a value of -35 mV was reached. Colored dots indicate mean for each voltage trajectory. (F) Plot of 1/first spike latency as a function of mean membrane voltage (mean of trajectories shown in F).

To better understand how Δ_*T*_ values determine the membrane voltage trajectory associated with both the initial and interspike interval spike approach, we used phase-plane plots to analyze the eLIF model using Δ_*T*_ values of 2 mV and 15 mV. In the phase-plane plot representation, the dashed lines indicate the membrane derivative function (*d*V/*d*t). The farther the value of the dashed line is from the x-axis at zero, the faster the membrane voltage changes. Through its effect on the shape of the *d*V/*d*t line, the Δ_*T*_ parameter determines the rate at which membrane voltage reaches spike threshold (Fig 7A and 7B). With Δ_*T*_ = 15 mV, the *d*V/*d*t function is shallow and generates small values, as indicated by the close proximity to the zero x-axis, for a large portion of the trajectory leading up to spike threshold; this results in a trajectory that changes more slowly and spends a small fraction of time in close proximity to spike threshold (Fig 7A). Reducing Δ_*T*_ increases *d*V/*d*t values for all voltages leading up to threshold. Under these conditions, the voltage trajectory only begins to slow in the immediate vicinity of spike threshold and, consequently, spends a large fraction of the interspike interval in close proximity to threshold (Fig 7A and 7B; insets).

The above analysis suggests that Δ_*T*_ influences fluctuation-based modulation of input-output functions at a given inter-spike interval value by setting the fraction of time that voltage spends in close proximity to spike threshold. As Δ_*T*_ becomes smaller, voltage trajectories spend an increasing fraction of time near the voltage for spike threshold, whereby small fluctuations can lead to spike events very early in the evolution of the trajectory. Conversely, by linearizing the trajectory, large Δ_*T*_ values limit fluctuation-induced spikes to time points later in the evolution of the voltage trajectory. To illustrate this, we quantified the probability that voltage fluctuations cause a spike at different time and voltage points during a single inter-spike interval trajectory (Fig 7C). Thus, each point along the trajectory provided initial conditions from which to calculate the likelihood of generating a spike at that given voltage and time point. From each of these points, we ran the models for a 50 ms period of time and calculated the probability of spike discharge in response to voltage fluctuations (SD = 2.5 mV) within this time period using 1000 trials (see Methods). As indicated in Fig 7C, for the majority of the interval, the likelihood of generating a spike in response to voltage fluctuations is substantially higher for Δ_*T*_ = 2 mV than that for Δ_*T*_ = 15 mV. Although a value of Δ_*T*_ = 15 mV leads to higher spike discharge probabilities towards the end of the interspike interval trajectory, the effect is limited in time and occurs late in the cycle, whereby the impact on spike rate relative to the deterministic case is small.

The influence of Δ_*T*_ on the *d*V/*d*t line also changes the scaling between mean membrane voltage and spike frequency. As indicated above, changes in Δ_*T*_ alter the *d*V/*d*t line, with a larger value of Δ_*T*_ generating a shallower *d*V/*d*t line. With the addition of positive (depolarizing) current, the *d*V/*d*t line moves upwards and away from x-axis, which results in a faster voltage trajectory (i.e. shorter interspike intervals).

When Δ_*T*_ = 2 mV, the voltage trajectory approaches values near threshold (*V*_*T*_) more quickly than with Δ_*T*_ = 15 mV. With Δ_*T*_ = 2 mV, the membrane derivative only slows down near the inflection point of the *d*V/*d*t line. Consequently, as the *d*V/*d*t line shifts upwards, the mean of the voltage trajectory remains largely unchanged because the majority of the trajectory is represented by the value near the inflection point of the *d*V/*d*t line (Fig 7E and 7F). In essence, a Δ_*T*_ = 2 mV compresses the voltage trajectory to a value approximately equal to *V*_*T*._ With Δ_*T*_ = 15 mV, however, a shift upwards in the *d*V/*d*t line significantly accelerates the voltage variable for a much larger fraction of the trajectory profile, especially for time points early in the trajectory that are far from the inflection point of the *d*V/*d*t line. As a result, the mean of value of the voltage variable depolarizes as the applied current magnitude increases and the *d*V/*d*t line shifts upwards (Fig 7E and 7F). By generating a shallow *f-V*, a large Δ_*T*_ limits the ability for a change in voltage brought about through random fluctuations to increase spike firing rate. Conversely, when Δ_*T*_ is small and the *f-V* relationship is steep, voltage fluctuations can give rise to a large change in spike firing rate (Fig 7D).

**Fig 8:**
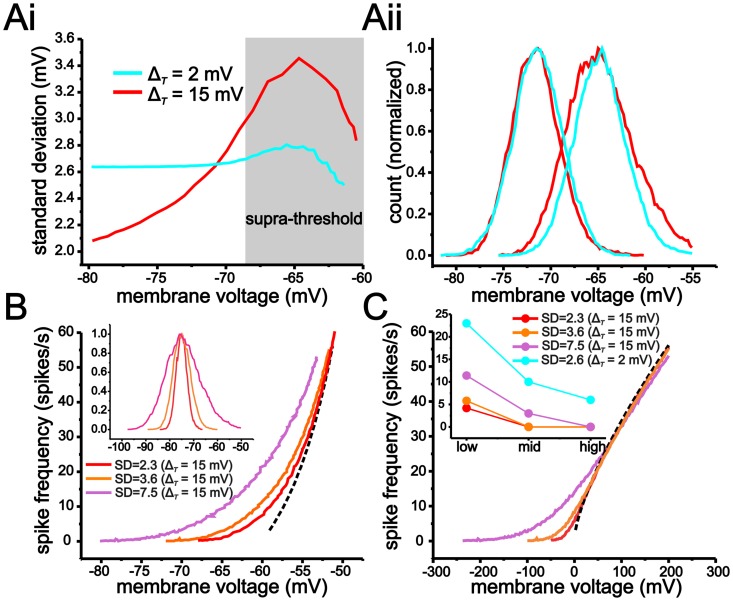
Increasing Δ_*T*_ has a greater impact on the scaling of the *f-V* relationship than the size of membrane voltage fluctuations. (A) Plot of the SD of membrane voltage fluctuations as a function of mean voltage using the eLIF model with Δ_*T*_ = 2 mV and Δ_*T*_ = 15 mV. The SD of membrane voltage fluctuations is larger with Δ_*T*_ = 15 mV for voltage values more depolarized than -70 mV due to the increase in membrane resistance. Histogram of membrane voltage fluctuations (Aii) in the eLIF model using a Δ_*T*_ = 2 mV and Δ_*T*_ = 15 mV at mean voltages of -71.5 mV and -68 mV. As shown, at -68 mV, the SD of membrane voltage fluctuations is greater (wider distribution) when Δ_*T*_ is set to -15 mV. (B, C) The shallow *f-V* relationship with Δ_*T*_ = 15 mV reduces fluctuation-based modulation of the *f-I* curve even when membrane voltage fluctuations are substantially larger than those when Δ_*T*_ = 2 mV. (B) Plot of *f-V* relationships generated with membrane voltage fluctuations with SD values of 2.3 mV, 3.6 mV and 7.5 mV (measured at -75 mV). Inset shows histogram of membrane voltage fluctuation for the three SD values. To increase the size of voltage fluctuations, the coefficient of current-input fluctuations was increased from 90 to 140 and 280. (C) Increasing the SD of membrane voltage fluctuations to 3.6 mV and 7.5 mV when Δ_*T*_ = 15 mV generates smaller changes in firing rate relative to Δ_*T*_ = 2 mV using voltage fluctuations with an SD of 2.6 mV. Inset shows plot of change in firing rate induced by voltage fluctuations for low, mid and high regions (defined as before) of the *f-I* relationship. Dashed black lines indicate *f-V* (B) and *f-I* (C) relationships in the absence of membrane voltage fluctuations.

With large Δ_*T*_ values, the increase in membrane resistance, and the resulting increase in voltage fluctuations, could potentially overcome the reduction in fluctuation-based modulation established by a shallow *f-V* relationship. Although the SD of membrane voltage fluctuations is larger in the peri-threshold region with Δ_*T*_ = 15 mV compared to Δ_*T*_ = 2 mV_,_ the difference is small compared to the changes in the *f-V* relationship established by increasing the value of Δ_*T*_ (Fig 8A). Thus, at the mean voltage where fluctuations first induce spiking in both models (~-68 mV), the SD of voltage fluctuations only increases from 2.64 mV to 3.06 mV when Δ_*T*_ is changed from 2 mV to 15 mV. In comparison, changes in the *f-V* relationship are much greater. With Δ_*T*_ = 2 mV, a 60 spikes/s range occurs entirely within a 0.5 mV range, but increases to a 10 mV range when Δ_*T*_ = 15 mV (Fig 7D). As a result, increasing Δ_*T*_ from 2 mV to 15 mV has a much larger effect on the scaling of the *f-V* relationship than the size of membrane voltage fluctuations. Even under conditions where the SD of membrane voltage fluctuations is increased to 3.6 mV or 7.2 mV (by increasing the current-input fluctuations), values much larger than those used with Δ_*T*_ = 2 mV, the modulation of the *f-V* and *f-I* relationships are still significantly less with Δ_*T*_ = 15 mV than with Δ_*T*_ = 2 mV (Fig 8B and 8C).

In summary, a large Δ_*T*_ generates voltage trajectories that spend a smaller fraction of time in close proximity to spike threshold and whose mean changes significantly with spike frequency. Both characteristics are the result of a gradual increase in membrane input resistance and help reduce modulation of input-output responses by voltage fluctuations.

### Reducing voltage-dependence of membrane resistance reduces fluctuation-based modulation of input-output curves in stellate cells

Our measures of input-resistance, voltage trajectories and analyses of the eLIF model generated two testable hypotheses regarding the sensitivity of stellate cell spike output to voltage fluctuations. Reducing the increase in input resistance associated with depolarization over the subthreshold region should lead to an increase in fluctuation-based modulation of input-output responses. This manipulation is akin to reducing the value of Δ_*T*_. Second, manipulations of membrane resistance using negative and positive sloped conductances should result in a reduction and increase, respectively, in fluctuation-based modulation of input-output responses, by manipulating the influence of the endogenous negative slope conductance and altering the voltage trajectories associated with the approach to spike threshold.

**Fig 9:**
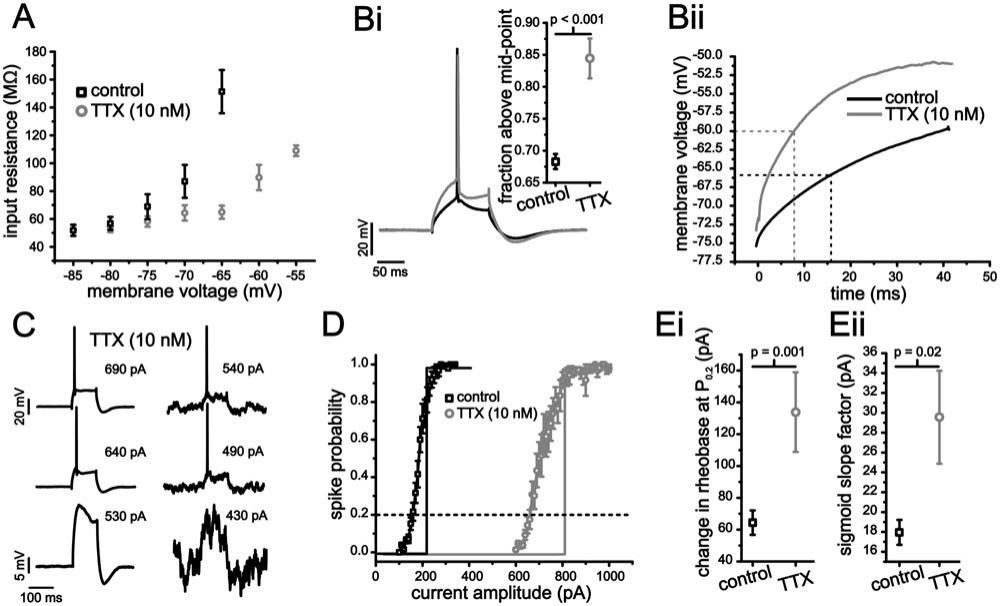
Block of voltage-dependent Na^+^ conductance increases sensitivity to membrane voltage fluctuations in stellate cells. (A) Steady-state membrane input resistance measures for different holding voltages under control (black squares) and with 10 nM bath applied TTX (grey circles). (B) Example voltage traces from a stellate cell in response to a 100 ms depolarizing current step (i), as well as the average for the first 50 ms (ii) under control (black) and with TTX (grey). Inset (Bi) shows the average fraction of time the voltage trajectory spent above the mid-point for control and under bath applied TTX. (C) Representative examples of stellate cell voltage response to 100 ms long current steps of different amplitudes with and without membrane voltage fluctuations under bath applied TTX. (D) Average spike-probability curves in the presence of membrane voltage fluctuations under control (black) and 10 nM TTX (grey). Vertical lines indicate rheobase in the absence of voltage fluctuations for each condition. (E) Plots of average leftward shift (i) and sigmoid slope factor (ii) for control (black squares) and TTX (grey circles).

Previous work in other neurons has established that steady-state Na^+^ conductance, mediated either by a window current or persistent Na^+^ conductance, can substantially increase membrane resistance with depolarization [26,41]. To establish the role of Na^+^ conductance in determining sub-threshold input resistance in stellate cells, we used a small concentration of TTX (10 nM) that was able to significantly alter sub-threshold membrane resistance but also maintain the ability to generate at least one spike. Application of TTX significantly reduced the gradual increase in input resistance across different voltages (2-way ANOVA, P <0.001, n = 12). At -65 mV, TTX reduced the input resistance from 151.4 ± 15.5 MΩ to 65.9 ± 4.8 MΩ (Fig 9A; P <0.001) without affecting the input resistance below -70 mV (Fig 9A; P >0. 32).

Next, we assessed the effect of Na^+^ conductance activation and a voltage-dependent increase in input resistance on the membrane voltage trajectory leading up to a spike. Because the membrane voltage trajectory to the first spike from a holding voltage of -75 mV could not be fit with an exponential function (Fig 2B and Fig 9B), we quantified changes in the first spike voltage trajectory by measuring the fraction of time spent above the mid-point of the trajectory. A trajectory with a large fraction above the mid-point is more similar to an exponential approach. The mid-point to threshold was calculated for each individual cell’s response to a square current step eliciting a ~50 ms latency to first spike. In the presence of TTX, the voltage trajectory spent a significantly larger fraction of time above the mid-point compared to control (Fig 9B; TTX: 0.84 ± 0.03 vs. control: 0.68 ± 0.01, P <0.001, n = 6–18). Unfortunately, 10 nM TTX eliminated the ability to generate continuous spike discharge that is required for *f-I* curve measures. As a consequence, we limited our analysis of cell output in the presence of TTX to spike-probability curves. As indicated, membrane voltage fluctuations were more effective at shifting the rheobase and smoothing the spike-probability curve in the presence of TTX (Fig 9D and 9E). The leftward shift in rheobase increased from 64.4 ± 7.7 pA to 134 ± 25 pA (Fig 9Ei; paired Student t-test, P = 0.001, n = 10), while the slope factor increased from 18.0 ± 1.3 pA to 29.6 ± 4.7 pA (Fig 9Eii; paired Student t-test, P = 0.02, n = 10). These results indicate that reducing the amount of sub-threshold Na^+^ conductance generates more exponential-like trajectories that result in increased fluctuation-based modulation of the spike-probability curves.

### Manipulation of membrane conductance using dynamic clamp alters voltage trajectories and modulation of input-output responses by voltage fluctuations

To further investigate the role of membrane resistance and negative slope conductance in shaping modulation of input-output response by voltage fluctuations, we manipulated membrane resistance in stellate cells using dynamic clamp by introducing artificial negative or positive slope conductances. In particular, we were interested in the effects of negative slope conductance since the ability for Na^+^ conductance to increase input resistance and slow the rate of change of membrane voltage is related to the negative slope associated with the Na^+^ *I-V* relationship [26]. Hence, the introduction of an artificial negative slope conductance should further decrease fluctuation-based modulation of input-output responses. Conversely, the addition of a positive slope conductance should increase modulation via voltage fluctuations by decreasing the influence of the endogenously expressed negative slope conductance.

For the negative slope conductance, we used a value of -5 nS, which was the maximum amount that could be added without introducing instabilities and that increased membrane resistance measured at -75 mV from 68.9 ±8.9 MΩ to 102 ± 13 MΩ. The positive conductance was set to 15 nS and decreased membrane resistance to 34.1 ± 2.7 MΩ. For the positive conductance, values greater than 15 nS led to a loss of continuous spiking in fashion similar to what has been reported in CA1 pyramidal cells using this manipulation [42,43]. Both the negative and positive conductances were linear with a reversal potential of -75 mV.

**Fig 10:**
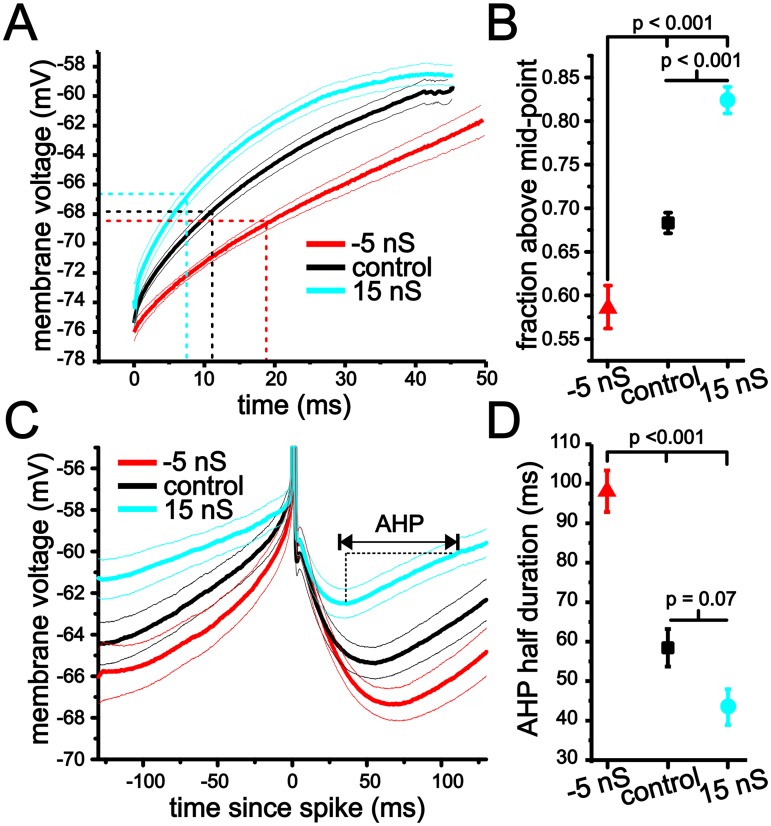
Changes in initial spike and interspike interval voltage trajectory using dynamic clamp. (A) Average membrane voltage trajectories for initial spike approach in stellate cells under -5 nS (red), control (black) and 15 nS of artificial membrane conductance added with dynamic clamp. Finer lines indicate sem. (B) Plot of average fraction above mid-point for initial spike voltage trajectories using -5 nS, control and 15 nS levels of artificial membrane conductance. (C) Average membrane voltage trajectories in stellate cells using dynamic clamp with -5 nS (red), control (black) and 15 nS (blue) of artificial membrane conductance. (D) Plot of average AHP half duration for the interspike interval voltage trajectories under -5 nS, control and 15 nS of artificial membrane conductance.

As with TTX experiments, we quantified changes in the first spike voltage trajectories using the fraction of time above the mid-point. Changes in membrane conductance had a significant impact on the trajectory leading up to spike threshold (Fig 10A; one-way ANOVA, P <0.001, n = 8–18). Negative conductance decreased the fraction of time above the midpoint to 0.59 ± 0.02, while adding positive conductance increased this this value to 0.82 ± 0.02 (Fig 10B; P <0.001, Tukey’s test).

Changing membrane conductance also had a significant impact on the duration of the AHP associated with continuous firing at ~4 Hz (Fig 10C; one-way ANOVA, P <0.001, n = 9–12). Negative conductance led to a significant increase in the AHP half-duration (time from trough to midpoint voltage between trough and spike threshold, Fig 10D; 98.1 ± 5.3 ms, P <0.001, Tukey’s test). Although positive conductance did not significantly alter the AHP half duration relative to control when taking repeated measures into account, there was a decrease in mean values (43.4 ± 4.5 ms, P = 0.07, Tukey’s test). Hence, overall, changes in membrane conductance altered the duration of membrane voltage trajectories leading to spike threshold in a form consistent with our analysis of the eLIF model.

**Fig 11:**
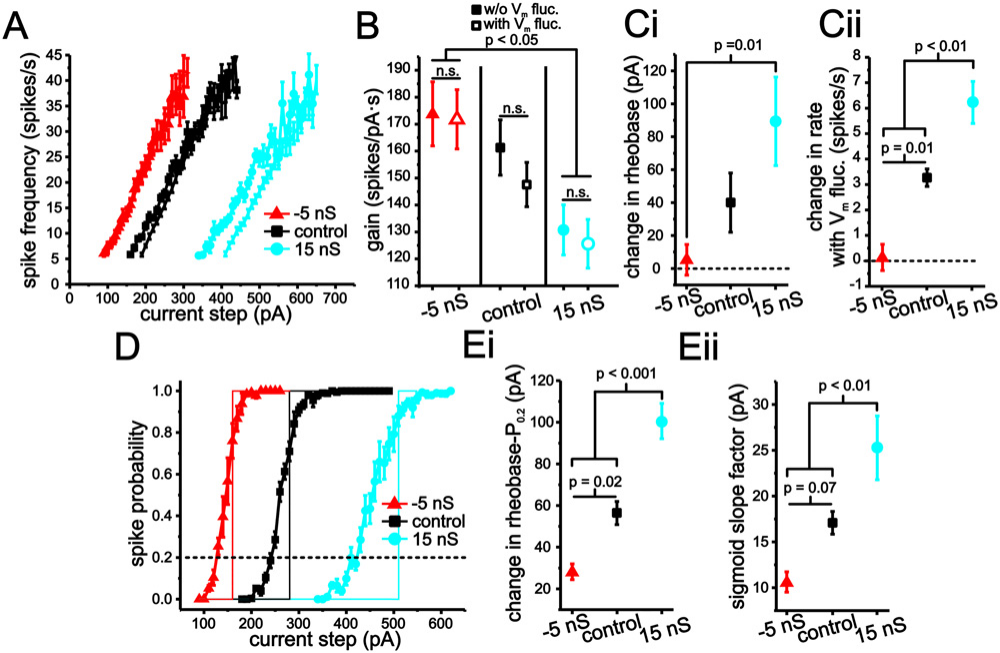
Modulation of stellate cell input-output responses with artificial changes in membrane conductance implemented using dynamic clamp. (A) Plot of average stellate cell *f-I* curves using -5 ns (red), control (black) and 15 nS (blue) levels of artificial conductance, as well as in the presence and absence membrane voltage fluctuations. (B) Plot of average gain measured using linear regression using -5 nS, control and 15 nS with (open symbols) and without (closed symbols) membrane voltage fluctuations. (C) Change in rheobase (i) and initial spike rate (ii) in stellate cell *f-I* curves resulting from the introduction of membrane voltage fluctuations with -5 nS, control and 15 nS levels of added conductance. (*D*) Plot of average stellate cell spike-probability curves under -5 ns (red), control (black) and 15 nS (blue) with and without membrane voltage fluctuations. (E) Change in rheobase (i) and sigmoid slope factor (ii) in stellate cell spike-probability curves under -5 nS, control and 15 nS resulting from the introduction of membrane voltage fluctuations.

Next, we quantified the effects of negative and positive changes in membrane conductance on fluctuation-based modulation of stellate cell input-output curves. For each conductance level, we compared changes in the slope and rheobase of *f-I* and spike probabilities curves induced by the introduction of membrane voltage fluctuations. As before, membrane fluctuations were kept at a SD of ~ 2.5 mV (-5 nS, 2.3 ± 0.1 mV, control: 2.41 ± 0.1 mV; 15 nS: 2.35 ± 0.01 mV, n = 5, 19, 18). Analysis of *f-I* curves indicated that gain was significantly modulated by changes in membrane conductance, but not by the introduction of membrane voltage fluctuations (Fig 11A and 11B; 2-way ANOVA, P <0.001 for conductance, P = 0.36 for voltage fluctuations). Contrary to expectations [8,9,44,45], *f-I* curve gain can be modulated, albeit modestly, by changes in membrane conductance only. More importantly, stellate cells *f-I* curves maintained a low degree of fluctuation-based modulation at all three conductance levels. We should note that eLIF model using a Δ_*T*_ = 15 mV, but not Δ_*T*_ = 2 mV, also generates a small decrease in gain when membrane conductance is increased. This result is related to changes in mean interspike interval membrane voltage induced by changes in conductance when Δ_*T*_ = 15 mV, but not Δ_*T*_ = 2 mV.

We proceeded to measure changes in rheobase and initial firing rate associated with *f-I* curves resulting from voltage fluctuations under each of the conductance conditions. Changes in membrane conductance significantly impacted the ability of artificial fluctuations to change rheobase (Fig 11Ci; P <0.02, one-way ANOVA, P = 0.01, Tukey’s test). Further, voltage fluctuations were not able to significantly reduce rheobase values in the presence of -5 nS conductance (Fig 11Di; one sample Student t-test, P = 0.58), while with 15 nS rheobase significantly increased (Fig 11Di; one sample Student t-test, P <0.001). As with rheobase, changes in the initial spike firing rate were also significantly changed by membrane conductance (Fig 11Dii; P = 0.001, one-way ANOVA), with each conductance level generating significant differences in initial spike firing rates (Fig 11Cii; P <0.01, Tukey’s test). With -5 nS conductance voltage fluctuations were not able to significantly increase firing rate from zero (Fig 11Cii; one sample Student t-test, P = 0.79), while these changes were significant under control and with 15 nS (Fig 11Cii; one sample Student t-test, P <0.001).

Fluctuation-induced changes in spike-probability curves mirrored those observed in the *f-I* curves (Fig 11D and 11E). For both rheobase and slope factors, conductance had a significant impact (Fig 11Ei and 11Eii; P <0.001, one-way ANOVA). Increasing membrane conductance by 15 nS led to both an increase in the ability for voltage fluctuations to shift the rheobase and smooth the spike-probability curves (Fig 11E). With a decrease in membrane conductance (-5 nS), rheobase changes and smoothing were less pronounced (Fig 11D and 11E).

Overall, these results are consistent with our hypothesis and indicate that a slower and more linear voltage trajectory, established by an increase in membrane resistance through a negative slope conductance, reduces modulation of input-output response by voltage fluctuations. Additionally, the linearization of voltage trajectories is fundamentally related to the negative slope conductance associated with Na^+^ current activation. Our data show that non-linear membrane properties shape the potential for voltage fluctuations to modulate the input-output responses of neurons.

## Discussion

Our study demonstrates results that have important implications for single-cell input-output modulation via changes in membrane conductance and voltage fluctuations. First, stellate cell input-output responses are modulated to a small degree by membrane voltage fluctuations under control conditions, as well as with increased membrane conductance. Second, in both models and stellate cells, a voltage-dependent inward current resulting from a negative slope conductance that activates over a significant sub- and peri-threshold region is responsible for low levels of fluctuation-mediated modulation of input-output responses. Finally, negative slope conductance reduced fluctuation-mediated changes in input-output response via a linearization and slowing-down of membrane voltage trajectories that also established a shallow and non-linear *f-V* curve.

### Controlling neuronal spike output with membrane voltage fluctuations and changes in membrane conductance

Membrane voltage fluctuations and changes in membrane conductance (e.g. shunting inhibition) are believed to be key factors that control neuronal input-output scaling [7–9,11,45]. A generally accepted role of voltage fluctuations is to lower spike threshold and amplify weak inputs [5,6]. Thus, the size and spectral content of voltage fluctuations can be used to potentially gate inputs, as suggested by some network models [46]. In addition, fluctuating current input can divisively scale the *f-I* curve by disproportionately increasing spike firing rate near threshold [5,7–10]. Moreover, under fluctuation-driven spiking, it has been shown that spike firing rate can scale with sub-threshold membrane voltage for a meaningful portion of a cell’s dynamic range [9,10]. For this reason, in the presence of significant voltage fluctuations, changes in the sub-threshold *I-V* relationship brought about through shunting inhibition, balancing excitatory and inhibitory conductances, or simply introducing a leak conductance are expected to translate into a further reduction in the slope of the *f-I* curve. Changes in the slope of the *f-I* curve are believed to be critical for setting the tuning curve of individual cells and have been proposed to play a critical role in sensory processing, particularly in the visual system with regards to setting the neuronal spike response to contrast [3,12,37,45]. Synaptic-mediated voltage fluctuations have therefore been implicated in setting both neuronal spike threshold and the overall scaling of the input-output relationship.

Data from cerebellar granule cells [9] and simulations from compartmental models [10] has convincingly demonstrated that spike rate can indeed scale with sub-threshold membrane voltage once voltage fluctuations are added. In granule cells, for example, fluctuations permit spike generation over a range of more than 100 spikes/s across what would otherwise be sub-threshold voltage values in the absence of fluctuations. Our data, however, suggest that this scenario is not generalizable, at least within the limits of physiological voltage fluctuations. In the case of stellate cells and our eLIF model (Δ_*T*_ = 15 mV), spike frequency scaling with sub-threshold membrane voltage is severely constrained due to a very shallow *f-V* relationship, which limits the spike frequency range in which voltage fluctuations can generate spikes. Consistent with our interpretation, granule cells have very steep *f-V* curves in the absence of significant membrane voltage fluctuations, with a 250 spikes/s range occurring over less than 2.5 mV [9]. Similarly, simulations supporting this mechanism [10] have been carried out in a compartmental model also expressing a steep *f-V* relationship (80 spikes/s over ~ 2.5 mV). In contrast, stellate cells and the eLIF model (Δ_*T*_ = 15 mV) generate *f-V* curves with slopes in the range of 4–5 spikes/mVs. Results presented here, therefore, indicate that the characteristics of the *f-V* curve and its relation to voltage trajectories leading up to spikes are crucial to understanding the degree that neuronal input-output functions are modulated by voltage fluctuations.

### Power-law scaling of stellate cell f-V curve without voltage fluctuations

In the visual system, a power-law scaling between spike firing rate and membrane voltage of layer II pyramidal neurons is critical for gain control and contrast invariance [12,37]. Modeling has shown that a power-law scaling with an exponent near 2 between spike firing rate and voltage can arise from the combination of an intrinsic, steep and linear *f-V* relationship, and smoothing through Gaussian-distributed voltage fluctuations [3,19,34,47]. In contrast with past assumptions, our data indicate that the *f-V* curve is not well approximated by a steep linear function, and that part of the non-linear scaling between spike rate and voltage can result from intrinsic voltage-dependent membrane properties.

For the eLIF model using a Δ_*T*_ = 15, the gradual activation of the negative slope conductance plays a critical role in setting the shallow, non-linear *f-V* curve. By activating gradually, depolarization results in a change of mean voltage at different spike discharge rates. This is because the rate of change in voltage is heavily influenced by the activation of the negative slope conductance. As greater amounts of the negative slope conductance are activated incrementally, the shape and mean of the interspike-interval voltage trajectory change considerably. This is not the case with Δ_*T*_ = 2 mV because the voltage trajectory at different frequencies is largely set by the passive properties of the membrane, which result in an exponential approach that does not experience a change in mean with increasing levels of depolarization and firing rate.

### Implications for MEC network function

Stellate cells have been implicated in spatial navigation via their grid-like spatial firing fields [48]. It is possible that the low degree of modulation of input-output responses by membrane voltage fluctuations in stellate cells helps maintain stable firing patterns with respect to spatial position. By reducing the influence of rapid voltage fluctuations on firing rate and input-output responses, stellate cell behavior likely promotes increased reliability to inputs associated directly with relevant network activity [31,32]. Consistent with this interpretation, our past work on spike-phase locking in stellate cells demonstrated a very high degree of spike-phase locking to slow (1–10 Hz) oscillatory inputs in the presence of random voltage fluctuations and increased membrane conductance [49].

### Role of Na^+^ conductance and threshold dynamics in determining input-output modulation by voltage fluctuations

A key factor in decreasing input-output modulation by membrane voltage fluctuations in stellate cells is the gradual activation of Na^+^ conductance. By slowing the approach to spike threshold, the gradual activation of Na^+^ conductance results in membrane voltage being farther from threshold for a large fraction of the trajectory. Current injections associated with *f-I* curve measures lead to a graded change in mean voltage and small, incremental increases in firing rate, resulting in a shallow *f-V* relationship. Both an LIF model and an eLIF model implemented with small Δ_*T*_ values express linear sub-threshold membrane properties. This leads to an exponential approach to threshold in which membrane voltage plateaus early and a large fraction of time is spent near spike threshold. As a result, small changes in current input and membrane voltage result in large changes in firing rate.

In addition to stellate cells, cortical neurons in the visual cortex [39], cerebellar Purkinje cells [50] and striatal interneurons [51] show a gradual increase in membrane resistance with depolarization that is mediated by Na^+^ current. Hence, the behavior observed in stellate cells is likely applicable to a wide range of different neurons.

From a non-linear dynamics perspective, an increase in membrane resistance that results in a region of negative slope conductance in the vicinity of threshold is consistent with a saddle-node bifurcation. This bifurcation is often associated with type I characteristics present in cortical pyramidal cells [52]. On the other hand, fast-firing interneurons in cortex have been classified as type II, with threshold behavior often modeled using a Hopf bifurcation and hence not requiring an increase in membrane resistance [52,53]. Previous modeling and experimental studies have suggested that type I behavior promotes a high sensitivity to membrane voltage fluctuations [18,52,54]. Unfortunately, drawing a clear relationship between the degree of modulation of input-output responses by voltage fluctuations and the type of threshold bifurcation is difficult. Our mechanism requires a graded increase in membrane resistivity over a 20 mV range, while a determination between a saddle-node and a Hopf bifurcation is established in the immediate vicinity of spike threshold, which is often less than 1 mV. In vivo voltage fluctuations, however, can span more than a 10 mV range such that the integration behavior of a cell over large regions of sub-threshold voltage, which are well outside the immediate vicinity of spike threshold, become crucial to understanding how a cell reacts to voltage fluctuations. For these reasons, we believe that either form of bifurcation and type can give rise to low or high sensitivity in input-output responses to random voltage fluctuations.

## Materials and Methods

### Ethics statement

All experimental protocols were approved by the University of Utah Institutional Animal Care and Use Committee.

### Tissue preparation

Horizontal sections of hippocampus and entorhinal cortex were prepared from 25 to 50 day-old Long-Evans rats of either sex. All chemicals were obtained from Sigma-Aldrich (St. Louis, MO) unless otherwise noted. After anesthetization with isoflurane and decapitation, brains were removed and immersed in 0°C artificial cerebrospinal fluid (ACSF) solution consisting of the following: (in mM): NaCl (125), NaHCO_3_ (25), D-glucose (25), KCl (2), CaCl_2_ (2), NaH_2_PO_4_ (1.25), MgCl_2_ (1), and buffered to pH 7.4 with 95/5% O_2_/CO_2_ gas. Horizontal slices were cut to a thickness of 400 μm (Leica VT 1200, Leica Microsystems; Wetzlar, Germany). After the cutting procedure, slices were incubated in ACSF at 30°C for 20 minutes before being cooled to room temperature (20°C). After the incubation period, slices were moved to the stage of an infrared differential interference contrast-equipped microscope (Axioscope 2+; Zeiss, Oberkochen, Germany). All recordings were conducted between 32 and 34°C.

### Electrophysiology

Electrodes were drawn on a horizontal puller (P97; Sutter Instruments, Novato, CA) and filled with an intracellular solution consisting of the following (in mM): K-gluconate (120), KCl (20), HEPES (10), diTrisPhCr (7), Na_2_ATP (4), MgCl_2_ (2), Tris-GTP (0.3), EGTA (0.2) and buffered to pH 7.3 with KOH. Final electrode resistances were between 2 and 5 MΩ, with access resistance values between 5 and 16 MΩ. Bridge balance compensation was used for all recordings. Seal resistance values were always greater than 1 GΩ. Electrophysiological recordings were performed with a current-clamp amplifier (Axoclamp 2B; Molecular Devices, Union City, CA), and data were acquired using custom software developed in Matlab (v. 2011, Mathworks, Natick, MA) utilizing the data acquisition toolbox. An estimated junction potential of 10 mV was subtracted for data analysis. Thus, the average resting potential reported here (-75 mV) is 10 mV more hyperpolarized than those reported elsewhere for stellate cells [55,56].

Stellate cell identity was established using the following criteria: 1) presence of a hyperpolarization-mediated membrane voltage sag, 2) impedance and resonance measures indicating a steady-state input resistance at rest between 35 MΩ and 80 MΩ and the presence of a ~5 Hz resonance peak determined using methods described previously [49] and 3) the location and cell morphology under DIC-IR optics (i.e. in layer II of MEC and with a non-pyramidal cell body shape).

For voltage clamp experiments, we held cells at each corresponding voltage (-85 to -65 mV) and used a small step (5 mV) and measured the change in current. The ratio of the change in voltage and current was used to measure input resistance at each corresponding holding voltage.

For dynamic clamp experiments, the current-clamp amplifier was driven by an analog signal from an x86 personal computer running Real-Time Application Interface Linux and Real-Time eXperimental Interface (RTXI)[57,58]. Shunting inhibition (*I*_*inh*_) was implemented using RTXI with the following equation:

$$I_{inh}\;=\;g_{inh}(V-E_{inh})$$

For these experiments, *E*_*inh*_ and *g*_*inh*_ were set to -75 mV and 15 nS, respectively. In the case of negative conductance (Fig 10 and 11), the *g*_*inh*_ term was set to -5 nS. For all experiments, the sample rate of the dynamic clamp was set to 10 kHz. A measured junction potential of approximately 10 mV was subtracted from all recordings. Data were collected at 10 kHz and filtered at 3 kHz. Current input fluctuations were implemented with filtered white noise using a low pass (*f*_*cut*_= 100 Hz) filter. The current signal was constructed in the frequency domain using a frequency amplitude (*A*(*f*)) scaling of *A*(*f*) = 1/(1+(*f*/*f*_*cut*_)). Matlab’s *ifft* function was used to implement an inverse Fourier transform and generate the time series from signals constructed in the frequency domain. For each cell, we recorded a short trial period in which the current input fluctuations were adjusted to maintain a standard deviation (SD) in membrane voltage fluctuations at rest (-75 mV) of ~2.5 mV.

For Na^+^ channel block, tetrodotoxin (TTX, Tocris, Bristol, UK) was bath applied at a concentration of 10 nM. Recordings with TTX were carried out approximately 15 minutes after bath application of the drug.

### Simulations and models

For the exponential leaky integrate-and-fire (eLIF) model [27], membrane voltage dynamics were governed by the following differential equation:

$$C\frac{dV}{dt}\;=\;I_{e}+g_{L}\Delta_{T}e^{\frac{V-V_{T}}{\Delta_{T}}}-g_{L}(V-E_{L})$$

where *C* = 170 *pF*, *V*_*T*_ = -60 *mV*, *g*_*L* =_ 25 nS, E_L_ = -75 mV and Δ_*T*_ = 15 mV (default). Note, for the passive version of the model (i.e. standard leaky integrate-and-fire), the first *g*_*L*_ term was set to zero and a separate leak term was used with the same reversal potential and using a conductance value of 15 nS. This value of conductance generates a passive model with the same input resistance as the eLIF model at -75 mV. Because of the exponential term in the eLIF, membrane voltage diverges to infinity upon crossing V_*T*_. For the eLIF simulations, membrane voltage was reset to V_*R*_ (-65 mV) upon reaching a value of 0 mV. The passive model lacks a true threshold phenomena, therefore an artificial threshold was set at -55 mV and membrane voltage was reset to -65 mV after crossing this threshold value. All models were simulated in Matlab and solved with a forward Euler method using a time step of 0.01 ms. We also tested model solutions with a time step of 0.001 ms and found the same results.

For the H-H formulism-based model (Fig 6), membrane voltage was governed by the following equations:

$$C\frac{dV}{dt}=I_{e}-g_{Na}m\left(1-n\right)\left(V-E_{Na}\right)-g_{Nap}p\left(V-E_{Na}\right)-g_{K}n\left(V-E_{Na}\right)-g_{L}\left(V-E_{L}\right)$$

dndt=(n∞−n)τn,(K+conductance)

m=1(1+eV+30−3),(transientNa+conductance)

n∞=1(1+eV+30−3),(K+conductancesteady-stateactivation)

p=1(1+eV−15),(persistentNa+conductance)

where *C* = 170 pF, *τ*_*n*_ *= 3* ms, *E*_*Na*_ = 50 mV, *E*_*K*_ = -90 mV, *E*_*L*_ = -80 mV, *g*_*Na*_ = 170 nS, *g*_*K*_ = 90 nS, *g*_*Nap*_ *= 150* nS and *g*_*leak*_ = 20 nS. To reduce the dimensionality of the model we used the approximation that *h*≈*1-n* and that both *m* and *p* equilibriate with membrane voltage instantaneously due to their time constants being smaller than the membrane time constant.

As with experiments, current fluctuations were generated using filtered white noise generated using the same equation and cut-off frequency (100 Hz) as in experiments. To ensure a SD of 2.5 mV at a voltage of -75 mV, each model was tested with incrementally larger noise coefficients until a SD of 2.5 mV was reliably measured over a small region of coefficient values at -75 mV over a 15 s duration. Current fluctuations were added to the DC current term (*I*_*e*_) in the above equation (i.e. additive noise). To increase model membrane conductance, a separate *g*_*L*_term was added using a reversal potential of -75 mV.

### Analysis and statistics

All analyses were carried out in Matlab using custom software and/or built in functions. For power-law and Boltzmann fits, we also confirmed fits in Origin 8.5 (OriginLab, Northampton, MA). Spike times were determined using a threshold crossing for membrane voltage. Spike frequency was determined using the mean inverse of the first three inter-spike intervals elicited from one or two second current steps. Average gain values were determined by averaging the individual slope values attained using a linear regression analysis of the *f-I* relationship. Rheobase values were calculated as the minimal current required to elicit 4 spikes from a holding voltage of -75 mV. For illustrative purposes concerning the average *f-I* curves shown in Fig 1 and 8, we calibrated the starting point of the *f-I* curve such that each cell’s initial value was near the average rheobase calculated for a given condition. For spike-probability curves, the current step duration was 100 ms and each current step size was repeated 15 to 25 times, with spike-probability defined as the number of steps that evoked spikes divided by the total number of steps. Individual spike-probability (*P*(*I*)) curves were fit with a Boltzmann function as follows:

P(I) = 11+eI-Ihalf-k

where *P* is the probability of spike discharge,*I*_*half*_is the current step size value required to elicit 0.5 probability (*P*_0.5_) in spike discharge and *k* is the slope (larger values denote a shallower slope) of the curve. Fits were used to calculate the slope factor (*k*), while the rheobase for probability curves was defined as the current step size required to elicit a *P*_0.2_ in spike discharge.

For the *f-V* curve, experimental and modeling results were fit using a power-law function:

$$f\left(V\right)\;=\;{a\left|V-V_{c}\right|}^{p}+b$$

where *f* is the firing rate, *p* is the exponent of the fit reported in the results section, *a* and *b* are positive constants and *V*_*C*_ is the minimal voltage required to elicit spike generation. Note that the term *a* was bounded such that only values of 1 or greater were possible. All fits used a least-squares method.

To quantify the effect of membrane voltage fluctuations on spike discharge in models during continuous spiking (Fig 7C), we first considered the model cell spiking periodically in the absence of random fluctuations (with period of ~270 ms), and defined the interval fraction as the inter-spike interval time divided by the total period. At every given fraction of the interval, we took the deterministic state of the model (value for voltage and all other variables) and used it as the initial condition for a set of 1000 test simulations, each 50 ms long in which fluctuations were then added. We defined probability as the number of simulations generating a spike divided by the total number of simulations at each given interval fraction. Thus, our probability measures how likely it is that voltage fluctuations will introduce one spike at a given phase and provides a measure for the ability of voltage fluctuations to change the spike rate associated with the low current region of the *f-I* curves.

For multiple comparisons, statistical significance was determined using either a one-way or two-way ANOVA. For repeated measures of means, statistical difference was determined using Tukey’s honestly significant criteria, while a Student t-test (one- or two-sample) was used for comparison of one or two values. Means are presented along with the standard error of the mean (s.e.m.).
